# A spatio-temporal analysis to identify the drivers of malaria transmission in Bhutan

**DOI:** 10.1038/s41598-020-63896-7

**Published:** 2020-04-27

**Authors:** Kinley Wangdi, Zhijing Xu, Apiporn T. Suwannatrai, Johanna Kurscheid, Aparna Lal, Rinzin Namgay, Kathryn Glass, Darren J. Gray, Archie C. A. Clements

**Affiliations:** 1 Department of Global Health, Research School of Population Health, Australian National University, Acton, Canberra, ACT 2601 Australia; 2 Department of Parasitology, Faculty of Medicine, Khon Kaen University, Khon Kaen, Thailand; 3 National Centre for Epidemiology and Population Health, Research School of Population Health, Australian National University, Acton, Canberra, ACT 2601 Australia; 4 Vector-borne Disease Control Program, Department of Public Health, Ministry of Health, Gelephu, Bhutan; 5 Faculty of Health Sciences, Curtin University, Perth, WA Australia; 6 Telethon Kids Institute, Nedlands, Australia

**Keywords:** Malaria, Malaria, Epidemiology, Epidemiology

## Abstract

At a time when Bhutan is on the verge of malaria elimination, the aim of this study was to identify malaria clusters at high geographical resolution and to determine its association with local environmental characteristics. Malaria cases from 2006–2014 were obtained from the Vector-borne Disease Control Program under the Ministry of Health, Bhutan. A Zero-Inflated Poisson multivariable regression model with a conditional autoregressive (CAR) prior structure was developed. Bayesian Markov chain Monte Carlo (MCMC) simulation with Gibbs sampling was used to estimate posterior parameters. A total of 2,062 *Plasmodium falciparum* and 2,284 *Plasmodium vivax* cases were reported during the study period. Both species of malaria showed seasonal peaks with decreasing trend. Gender and age were not associated with the transmission of either species of malaria. *P. falciparum* increased by 0.7% (95% CrI: 0.3%, 0.9%) for a one mm increase in rainfall, while climatic variables (temperature and rainfall) were not associated with *P. vivax*. Insecticide treated bed net use and residual indoor insecticide coverage were unaccounted for in this study. Hot spots and clusters of both species were isolated in the central southern part of Bhutan bordering India. There was significant residual spatial clustering after accounting for climate and demographic variables.

## Introduction

Malaria continues to inflict a great health and socio-economic burden on humanity, with an estimated 3.2 billion people at risk of being infected^1^. In 2018, globally there were 228 million cases and 405,000 deaths, around 67% (272,000) of deaths were in children aged under 5 years^2^. However, in 2018, there were 23 million fewer cases as compared to 2010^2^. In 2016, malaria remained endemic in 91 countries and territories as compared to 108 in 2000^3^. The World Health Organization (WHO) African Region accounts for around 90% of malaria cases globally, followed by the South-East Asian Region (SEAR) (5%) and the Eastern Mediterranean Region (2%)^4^. Some of the factors that have led to the observed reductions in malaria incidence since 2000, are intensification of malaria control interventions supported by unprecedented financial support, socio-economic improvement in endemic countries and increasing urbanization^5–8^. In 2018, total investment for malaria control and elimination was US$ 2.7 billion^2^.

WHO developed the *Global Technical Strategy for Malaria 20*1*6–*2*0*3*0* (GTS)^5^ with an aim to fast track progress towards malaria elimination. This strategy is complemented by the Roll Back Malaria advocacy plan, *Action and Investment to Defeat Malaria 2016–2030* (AIM)^9^. GTS and AIM set a global goal to eliminate malaria in at least 21 countries by 2020, known as E-2020 countries and 35 countries by 2030^3,9^.

**Figure 1:**
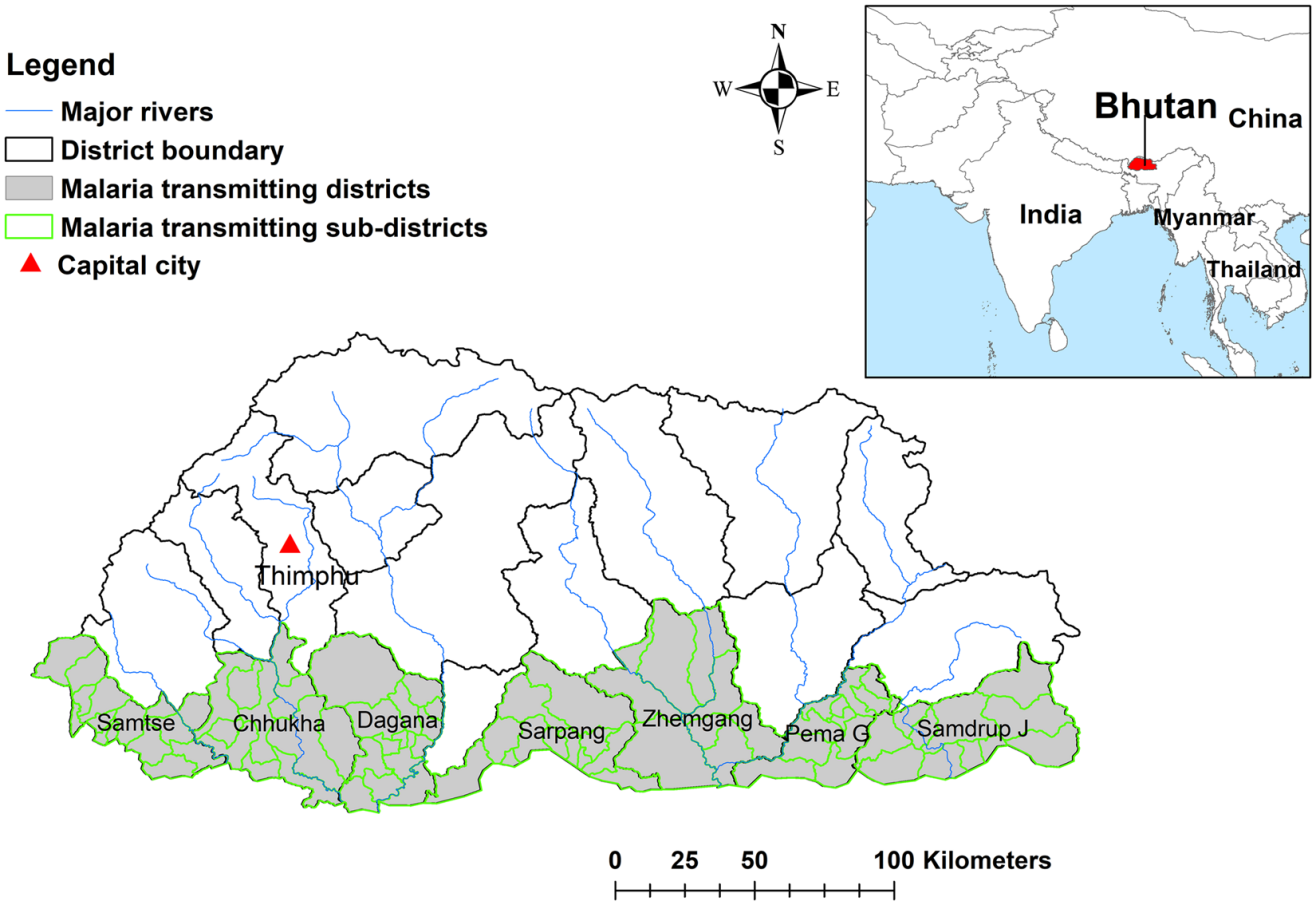
Map of Bhutan with malaria transmitting districts.

Malaria is reported from seven districts of Bhutan along the southern border with India. These districts are Chukha, Dagana, Pemagatshel, Samdrup Jongkhar, Samtse, Sarpang and Zhemgang (Fig. 1). Malaria control activities in Bhutan are based on: (1) Early diagnosis and prompt treatment with artemisinin-based combination therapy (ACT), (2) Protection of at-risk populations with long-lasting insecticide nets (LLINs) and indoor residual spraying (IRS), and (3) integrated vector management (IVM). With the dwindling of malaria cases, Bhutan was aiming to eliminate malaria by 2018^10^. However, there are still malaria cases and an updated elimination goal date has been set to 2020.

Small-scale geographic variation in transmission becomes increasingly important in the elimination phase as malaria cases are confined to hard-to-reach populations, often in border areas, that could act as source of reintroduction into areas which have achieved elimination^11,12^. Therefore, the aims of this study were to quantify the spatial and temporal patterns of malaria and associations between malaria risk and climatic factors. The information from this study can be used to intensify control measures to achieve malaria elimination goals in Bhutan.

## Results

### Descriptive analysis

**Figure 2:**
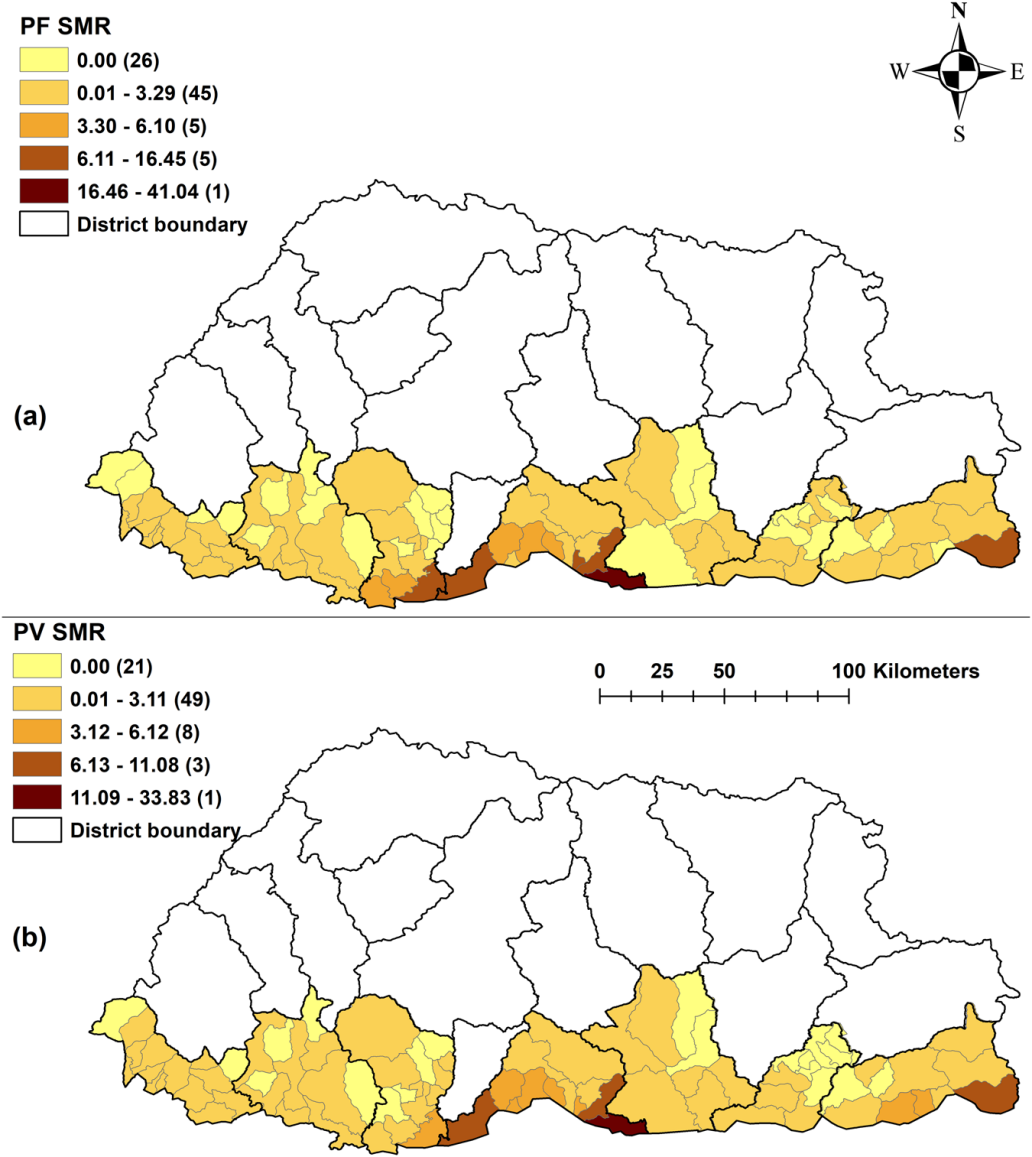
Raw standardised morbidity ratios of (**a**) *Plasmodium falciparum* and (**b**) *Plasmodium vivax* by sub-districts in Bhutan, 2006–2014.

A total of 2,062 *P. falciparum* and 2,284 *P. vivax* cases were recorded during the study period, of which 328 (8.2%) were mixed infection with both species. There were more cases amongst males older than 5 years than females and male children <5 years. In 2006, there were 1,721 cases and numbers subsequently reduced over the study period, with fewer than 20 cases in 2013 and 2014 respectively. There were no cases in the <5 years age group in 2013 and 2014 (Table 1). Maps of crude standardized morbidity ratios (SMR) of *P. falciparum* and *P. vivax* revealed higher risk in Samdrup Jongkhar and Sarpang districts, particularly in sub-districts adjacent to the international borders (Fig. 2).

**Table 1:** Malaria incidence stratified by sex and age group during the study period (2006–2014).

	*Plasmodium falciparum*						*Plasmodium vivax*					
	Male			Female			Male			Female		
	U5	5+	Total	U5	5+	Total	U5	5+	Total	U5	5+	Total
2006	23	520	543	14	240	254	39	566	605	30	289	319
2007	8	220	228	2	103	105	13	241	254	9	163	172
2008	3	102	105	6	48	54	7	97	104	2	48	50
2009	13	281	294	6	191	197	14	237	251	11	151	162
2010	4	94	98	2	52	54	3	159	162	5	82	87
2011	3	57	60	2	22	24	0	51	51	0	23	23
2012	1	16	17	1	14	15	2	17	19	0	11	11
2013	0	6	6	0	1	1	0	3	3	0	3	3
2014	0	4	4	0	3	3	0	6	6	0	2	2
Total	55	1,300	1355	33	674	707	78	1,377	1,455	57	772	829

U5- under 5 years; 5+- 5 years and older.

### Time-series decompositions

**Figure 3:**
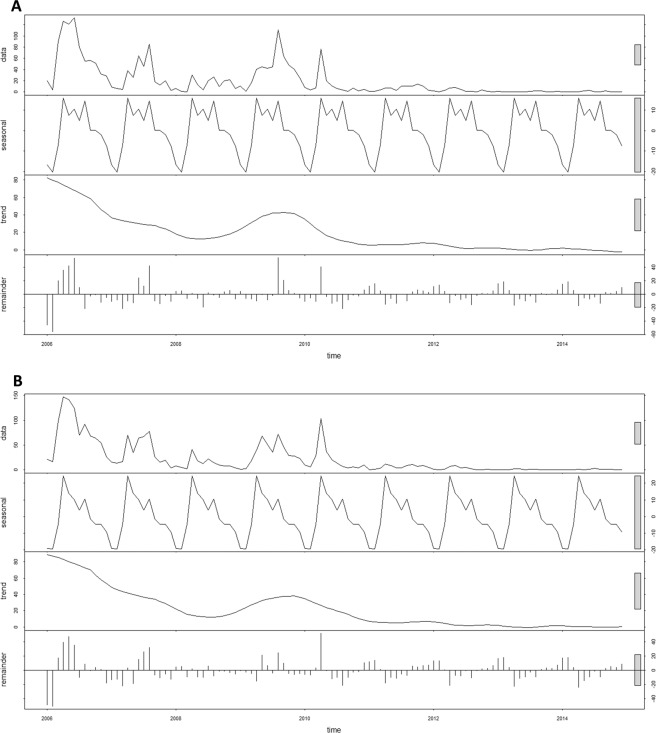
Decomposed *Plasmodium falciparum* and *Plasmodium vivax* of Bhutan, 2006–2014.

For both *P. falciparum* and *P. vivax*, time-series decompositions of raw data showed a clear seasonal pattern. Large peaks occurred for each parasite species in May of each year, with incidence dropping off during the remainder of the rainy season. The inter-annual pattern showed a large peak in 2006 and a small peak in 2010 with lower incidence in the intervening and subsequent years for both species of malaria (Fig. 3).

### Spatial autocorrelation analysis

**Figure 4:**
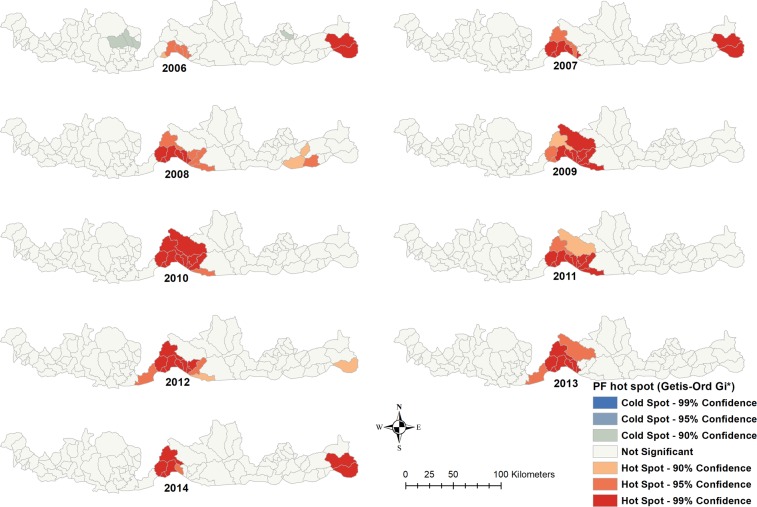
Time series hot spot analysis of *Plasmodium falciparum* of Bhutan, 2006–2014.

**Figure 5:**
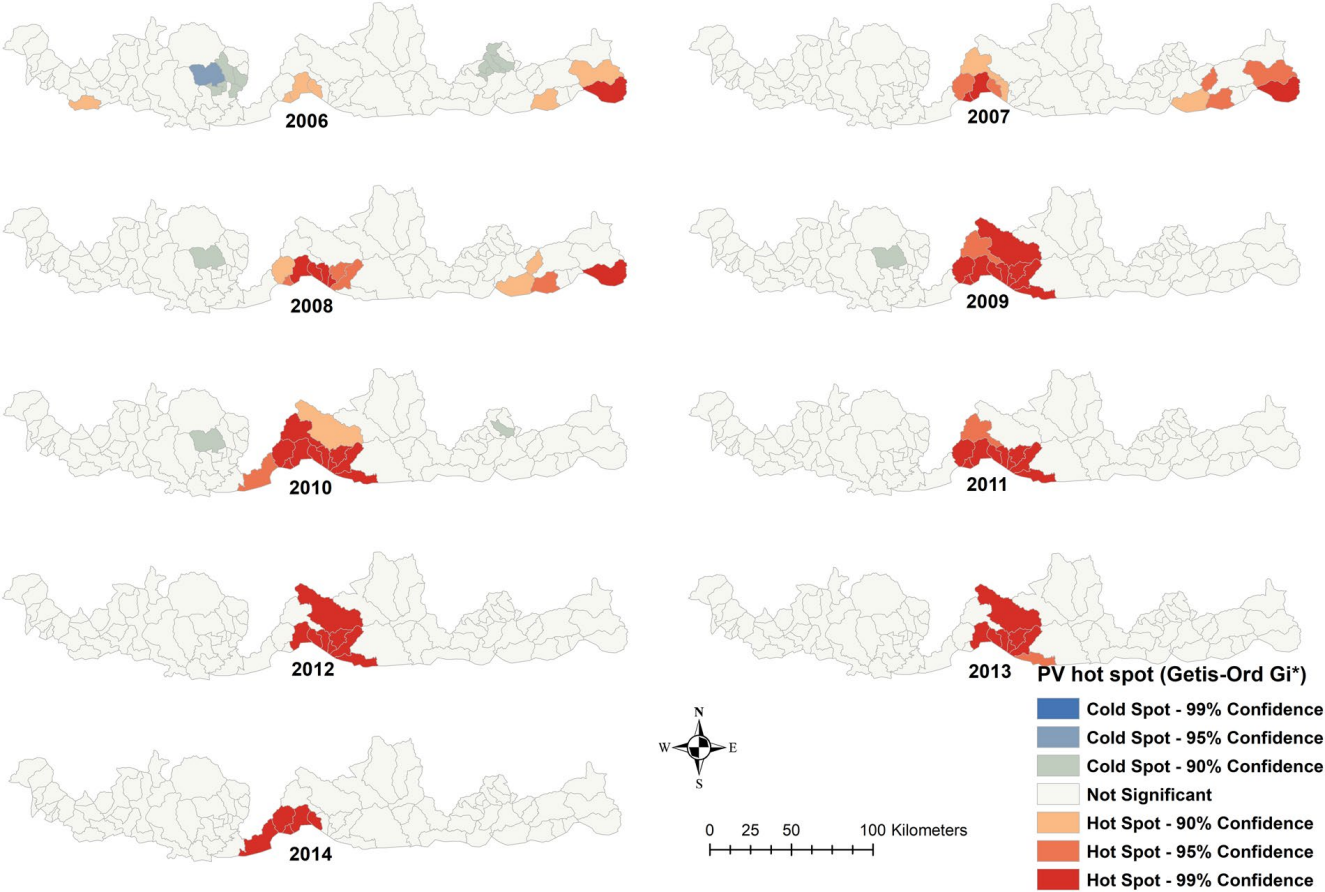
Time series hot spot analysis of *Plasmodium vivax* of Bhutan, 2006–2014.

Hot spots of both *P. falciparum* and *P. vivax* were reported in central and eastern parts of the Bhutan-India border region, while high-high clustering was located in the central part of the border region (Figs. 4 and 5**)**. Eight sub-districts in Sarpang were in high-high clusters for *P. falciparum* and *P. vivax*. Fifteen sub-districts were in low-low clusters in three districts of Chukha, Dagana and Pemagatshel for both *P. falciparum* and *P. vivax* (Supplementary Fig. 1).

### Spatio-temporal model

**Figure 6:**
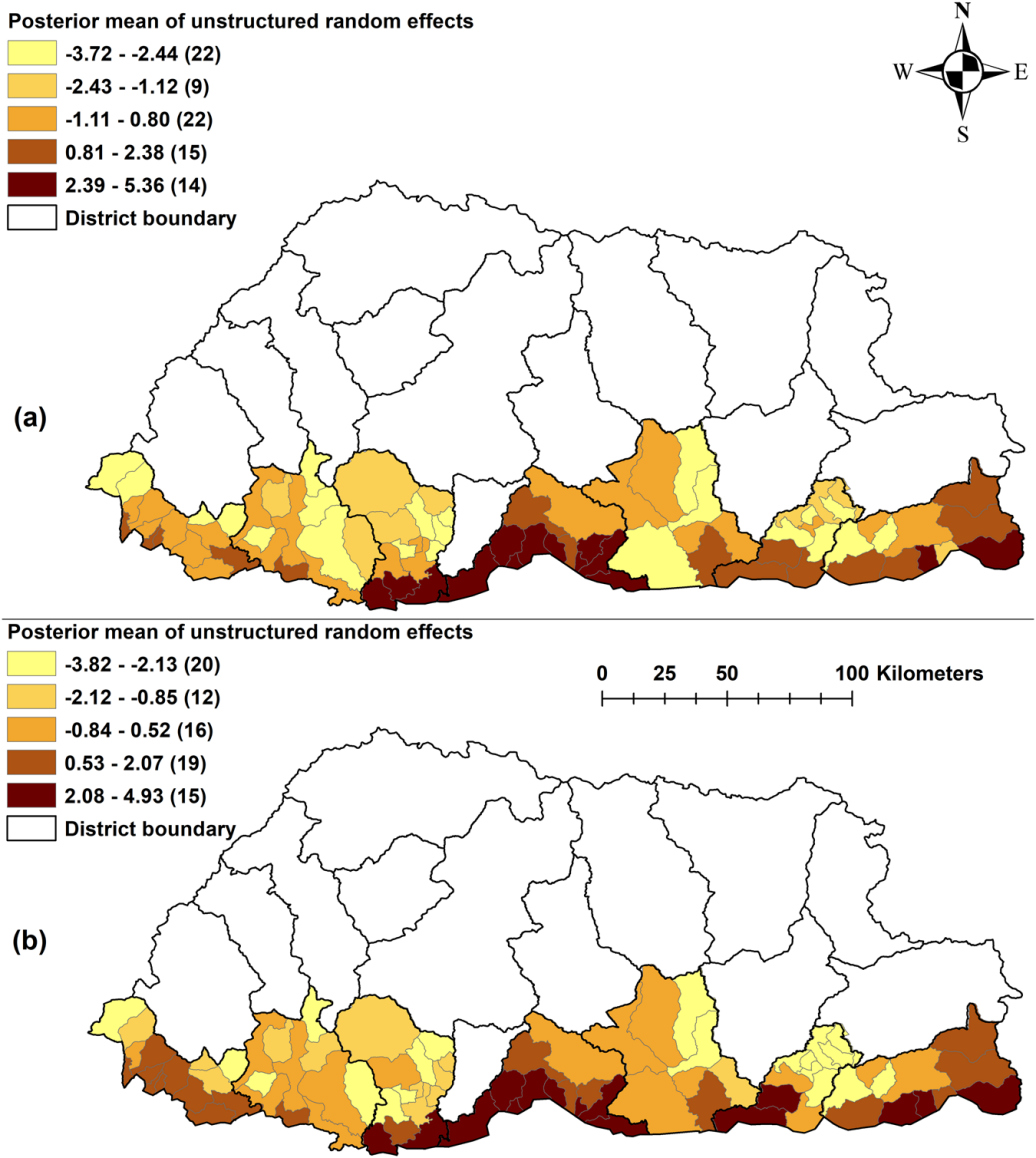
Spatial distribution of the posterior means of unstructured random effects for (**a**) *Plasmodium falciparum* and (**b**) *Plasmodium vivax* in Model I.

Table 2 describes the three spatio-temporal models: Model I, containing the unstructured random effects and Model III containing both the unstructured and structured random effects, had similar fit (indicated by a difference of deviation information criterion [DIC] of <3), and were better fitting than Model II containing the structured random effect only, for both *P. falciparum* and *P. vivax*. Here we present the results for Model I which is the simpler model. For *P. falciparum*, there was an estimated increase of 0.7% (95% credible intervals [CrI] 0.3%, 0.9%) for a one mm increase in rainfall. *P. falciparum* cases decreased by 2% (95% CrI 0.4%, 4.0%) for each 1 °C increase of maximum temperature, but this was not statistically significant. Similarly, a one mm increase in rainfall and 1 °C increase of maximum temperature was associated with a 0.2% increase (95% CrI −0.1%, 0.6%) and 0.1% increase (95% CrI −0.3%, 1.0%), respectively, in *P. vivax* incidence, but were statistically not significant. Sex and age were not statistically significant predictors of either species of malaria. The variance of the random effects for *P. falciparum* and *P. vivax* in this model were 0.16 (95% CrI 0.10, 0.24) and 0.20 (95%CrI 0.13, 0.29), respectively (Table 2). The means of the spatially unstructured random effects when mapped showed evidence of spatial clustering after accounting for the model covariates, despite these random effects not being spatially structured in the model (Fig. 6). Some sub-districts had a high probability of being above (or below) the overall mean residual risk for the study period (Supplementary Fig. 2). In general, these sub-districts matched the high-high and low-low clusters identified in the exploratory Local Indicators of Spatial Association (LISA) analysis.

**Table 2:** Regression coefficients and 95% CrI from Bayesian spatial and non-spatial models of *Plasmodium falciparum* and *P. vivax* cases reported by month and sub-districts, Bhutan, 2006–2014.

	*Plasmodium falciparum* RR (95% CrI)	*Plasmodium vivax* RR (95% CrI)
**Model I**^‡^		
Intercept^*^	−1.36 (−2.01, −0.78)	−1.07 (−1.74, −0.51)
Sex (reference group- male)	1.00 (0.81, 1.25)	0.98 (0.83, 1.17)
Age (reference group- <5 yrs)	1.00 (0.91, 1.10)	1.05 (0.96, 1.15)
Rainfall (mm)^†^	1.006 (1.003, 1.009)	1.006 (1.003, 1.006)
Temp Max (degree Celsius)	0.98 (0.96, 1.004)	0.99 (0.97, 1.01)
Probability of extra zero	0.26 (0.21, 0.30)	0.28 (0.23, 0.32)
Heterogeneity^*^		
Unstructured	0.16 (0.10, 0.24)	0.20 (0.13, 0.29)
Structured (spatial)		
DIC	7353	8573.17
**Model II**		
Intercept^*^	−1.47 (−2.11, −0.90)	−1.11 (0.01, −0.68)
Sex (reference group- male)	1.03 (0.61, 1.88)	1.00 (1.01, 1.48)
Age (reference group- <5 yrs)	1.01 (0.85, 1.19)	1.05 (1.00, 1.23)
Rainfall (mm)^†^	1.01 (1.00, 1.01)	1.00 (1.00, 1.03)
Temp Max (degree Celsius)	0.98 (0.95, 1.02)	0.99 (1.00, 1.02)
Probability of extra zero	0.26 (0.18, 0.33)	0.28 (0.23, 0.32)
Heterogeneity^*^		
Unstructured		
Structured (spatial)	0.05 (0.03, 0.07)	0.05 (0.00 0.08)
DIC	7415	8622.61
**Model III**		
Intercept^*^	−1.44 (−2.35, −0.73)	−0.99 (−1.57, −0.45)
Sex (reference group- male)	1.00 (0.79, 1.27)	0.98 (0.82, 1.17)
Age (reference group- <5 yrs)	1.00 (0.91, 1.11)	1.05 (0.96, 1.15)
Rainfall (mm)^†^	1.01 (1.00, 1.01)	1.00 (1.00, 1.01)
Temp Max (degree Celsius)	0.98 (0.96, 1.00)	0.99 (0.97, 1.01)
Probability of extra zero	0.26 (0.18, 0.33)	0.28 (0.23, 0.32)
Heterogeneity^*^		
Unstructured	0.17 (0.10, 0.36)	0.20 (0.13, 0.30)
Structured (spatial)	813.90 (0.10, 3,141.00)	576.70 (41.29, 2,105.00)
DIC	7356	8573.46

CrI- credible interval; DIC- deviation information criteria; RR- relative risk.

^‡^Best fit model ^*^co-efficient; ^†^One month lagged for *P. falciparum*.

## Discussion

This study aimed to describe the spatial and temporal epidemiology of malaria in the pre-elimination setting of Bhutan, which is aiming to eliminate malaria by 2020^13^. Time series decomposition showed seasonal peaks for both *P. falciparum* and *P. vivax*. Rainfall was associated with an increased risk of *P. falciparum*. Malaria clusters and hot spots of both species were located in Sarpang district.

Malaria transmission in Bhutan seems to have been interrupted and the risk of malaria is equal in both genders and across age groups (typical of low-transmission settings where there is little acquired immunity^14^). This finding is in line with an earlier cross sectional survey which showed zero prevalence^15^. Ongoing cases might relate to small localised clusters of transmission created by movement of infected individuals across the international border. Transmission interruption could be attributed to a number of factors including good coverage and use of LLINs^15,16^, prompt diagnosis and treatment and enhanced surveillance^17^.

Malaria transmission hot spots for both species continued to occur in the same region over 10 years^18^. Some of the reasons outlined for these hot spots were their proximity to a very porous border^16,19^ with the Indian state of Assam, which reported some of the highest malaria incidence rates in India^20–24^. However, there has been a significant reduction in the number of cases in Assam since 2016 as India has accelerated towards elimination by 2030^25^.

The seasonal peak of malaria corresponds well with the monsoon in Bhutan, which begins in June and lasts until September. Temperature plays a crucial role in the transmission cycle of the malaria parasite and mosquito survival^26,27^. Studies found that at a temperature of 22 °C, the life cycle of malaria parasite development in mosquito vector is completed in less than 3 weeks^28^. The biting rate and gonotrophic processes are also temperature dependent^29,30^. Other studies have reported rainfall as an important driver of malaria transmission^31,32^. However, the only climate association found in the current study was between rainfall and *P. falciparum* risk, perhaps reflecting the disruption to pre-intervention transmission dynamics caused by high LLIN and insecticide coverage in the study area.

*Plasmodium vivax* risk was not associated with any covariates in the model. A plausible reason could be that, unlike *P. falciparum*, *P. vivax* infection can hide in the liver, lying dormant (in hypnozoite form) and protected from the external environment until a relapse is activated. *P. vivax* represents the most frequent cause of malaria outside of Africa^33,34^. RDTs remain the cornerstone of diagnosis of *P. vivax* in many countries. However, RDTs are not adequately sensitive to diagnose the hypnozoite in the liver or in pregnant women. This makes it a particularly challenging parasite to identify and eliminate.

*P. vivax* is treated with cholorquine (three doses) and radical cure is done using primaquine over two-week period^34^. A recent study showed that choloroquine and primaquine are still effective against *P. vivax* in Bhutan^34^. Therefore, ascertaining adherence to the radical cure is crucial as Bhutan attempts to eliminate malaria by 2020. Not adhering to the treatment continues to be an important factor that leads to continued *P. vivax* transmission in other parts of the world^35,36^. It is recommended to undertake operational research to understand barriers and enablers to adherence to two weeks of radical cure of *P. vivax* in Bhutan to develop strategies to improve adherence.

The main strength of this study was the fine resolution of spatial analysis at the sub-district level over a long time series (108 months). However, there are some limitations that are worth noting. One of the major limitations in using surveillance data is that the completeness and representativeness of such data cannot be ascertained. Secondly, populations of sub-districts were projected and may have led to over or under estimation. Thirdly, there was no reconciliation to accommodate the different level of the climate variables (district) to the disease data (sub-district), and the climate conditions were assumed to be homogeneous within a district. Fourthly, all malaria cases were confirmed using microscopy. Even though conventional light microscopy is considered a “gold standard” for malaria parasite identification and confirmation, light microscopy has a detection limit of 5–50 parasites/μL^37,38^. Therefore, patients with low parasite intensity (<5 parasites/μL) could have been missed. Fifthly, *P. vivax* cases could have been inflated due to relapse because this was not accounted for when diagnosing *P. vivax*. Lastly, unmeasured risk modifiers, such as socio-economic development, living standards, treatment, localised behavioural patterns, population mobility, and bed net use and residual indoor insecticide coverage were unaccounted for in this study.

## Conclusion

*Plasmodium falciparum* transmission in Bhutan was associated with rainfall. Hot spots of both *P. falciparum* and *P. vivax* were isolated in sub-districts of Sarpang district. A high residual risk area of malaria transmission was identified in the same sub-districts. Targeted distribution of resources, including intensified interventions in this part of the sub-district will be required for local malaria elimination. Cross-border surveillance needs to be strengthened because of the risk of cross-border malaria to elimination and maintenance of elimination. Maps could be used to target surveillance.

## Methods

### Study site and data

Bhutan is located in the Eastern Himalayas, bordering China in the north and India in the east, south and west. The country is divided administratively into 20 districts and 205 sub-districts. In 2017, the total population of Bhutan was 681,720^39^. Historically, malaria transmission occurred in 82 sub-districts of seven districts with 209,090 people living in these districts (Fig. 1**)**.

In this study, malaria data of the seven districts from 2006 to 2014 were obtained from the national malaria surveillance system, hosted by the Bhutan Vector-borne Disease Control Program (VDCP). All other districts were assumed to be non-endemic and were excluded from the analysis. The dataset contained laboratory-confirmed malaria cases, defined as clinically diagnosed cases with either malaria parasite species, confirmed by microscopy. The infections were categorised by species: *P. falciparum* and *P. vivax* and stratified by gender and two age groups (<5 and ≥5 years) at the sub-district level.

Population estimates used in this study were from publications from the National Statistical Bureau and the Office of the Census Commissioner of Bhutan^40,41^. An electronic map of district boundaries in shapefile format was obtained from Global Administrative Areas database (http://www.gadm.org/country).

### Exploration of seasonal patterns and inter-annual patterns

The average monthly number of malaria cases was calculated from the full time series (January 2006-December 2014). The time series of malaria incidence was decomposed using seasonal-trend decomposition based on locally (STL) weighted regression to show: the seasonal pattern, inter-annual patterns and the residual variability. The STL model was structured as follows:

$${Y}_{t}={S}_{t}+{T}_{t}+{R}_{t}$$

where *Y*_*t*_ represents numbers of local malaria cases with logarithmic transformation, *S*_*t*_ is the additive seasonal component, *T*_*t*_ is the trend, *R*_*t*_ is the “remainder component” and *t* is time in months^42–45^.

### Spatial autocorrelation analysis

At a global (study area) scale, Moran’s I statistic was used to explore spatial autocorrelation and its strength and to test the assumption of spatial independence. Anselin Local Moran’s I statistic and Getis-Ord Gi* statistic were used to undertake local (sub-districts) level clustering and hot spots analysis^46^. Clustering is the occurrence of unusual aggregation of events in a sub-district. Hot spot is a form of clustering where the target sub-district reports higher rates than the study area average.

Anselin Local Moran’s I (LISA) was used to identify high-risk clusters, low-risk clusters and outliers (high-low and low-high). A positive Local Moran’s I (significant cluster) value shows that the target sub-district with high-risk cluster is surrounded by sub-districts with high-risk cluster or the target sub-district with low-risk cluster is surrounded by sub-districts with low-risk cluster. In a negative Local Moran’s I (outliers) value, the target sub-district with high-risk cluster is surrounded by sub-districts with low-risk or low-risk cluster is surrounded by high-risk cluster sub-districts^46^. Clusters are determined by comparing Moran’s I values of the target sub-districts and its neighbouring sub-districts to Moran’s I values of all sub-districts in the study area^46^. In significant high-risk cluster (*p*  ≤  0.05), the observed Moran’s I value is larger than the expected Moran’s I value. Where as for low-risk cluster, an opposite relationship is observed (observed Moran’s I is smaller than the expected Moran’s I value). In the case of spatial outliers (not significant) Moran’s I value remains in a neutral class^46^. These classifications of clusters were represented with sub-district boundaries. The Getis-Ord Gi* and Anselin Local Moran’s I cluster analytical methods were used to generate clusters and this served as a sensitivity analysis.

### Crude standardized morbidity ratios

Crude standardized morbidity ratios (SMRs) analyses of both species were undertaken to describe the malaria incidence by sub-districts across the study period (9 years). SMR was calculated from:

$${Y}_{i}=\frac{{O}_{i}}{{E}_{i}}$$

*Y*_*i*_*-*is the overall SMR in sub-district *i, O*_*i*_ - the total number of reported malaria cases in the sub-district *i* and *E*_*i*_*-* expected number of malaria cases in the sub-district *i*. The *E*_*i*_ was derived by multiplying the average population for sub-district *i* with the national incidence of malaria^12^.

### Independent variable selection

Best fit climatic covariates were selected using a Poisson regression and Akaike’s information criterion (AIC). Climatic variables (maximum and minimum temperature and rainfall) without a lag, and with one and two-month lag times, plus altitude, were fitted into univariate Poisson regression models. Maximum temperature, unlagged rainfall and altitude showed the best fit with the lowest AIC values. However, maximum and minimum temperature were found to be highly co-linear when tested using variance inflation factors (VIF). Therefore, minimum temperature was dropped from the final model, which included variables rainfall and maximum temperature.

### Spatio-temporal model

The number of zero counts for *P. falciparum* and *P. vivax* was 34,142 (96.4%) and 34,340 (96.9%). Therefore, we ran an analysis to determine the best model. Zero-inflated Poisson (ZIP) regression was selected over the standard Poisson regression because ZIP had a better fit with lower AIC and BIC as compared to Poisson regression and a Vuong test showed the two models were statically different (Supplementary Tables 1–4). Bayesian statistical software WinBUGS version 1.4 (Medical Research Council, Cambridge, UK and Imperial College London, UK) was used to run ZIP regression models for *P. falciparum* and *P. vivax*. Three different models were tested for each species; first model included only climatic variables (rainfall and maximum temperature) as explanatory covariates. Second model contained spatially structured random effects. Third model contained both climatic covariates and spatially structured random effects. The model with the lowest DIC was selected as the final explanatory model for each species.

The most comprehensive model, which had an outcome as the observed counts of malaria, *Y*, for *i*^th^ sub-district (*i* = 1…82) in the *j*^th^ month (January 2006-December 2014), age group *k*, and sex group *l* was structured as follows:

$$P({Y}_{ijkl}={y}_{ijkl})=\{\begin{array}{c}\omega +1(1-\omega ){e}^{-\mu },\,{y}_{ijkl}=0\\ (1-\omega ){e}^{-\mu }\,{\mu }_{ijkl}^{{y}_{ijkl}}/{y}_{ijkl},\,{y}_{ijkl} > 0;\end{array}$$

*Y*_*ijkl*_   ~Poisson (μ_*ijkl*_)

log (μ_*ijkl*_) = log(E_*ijkl*_) + *θ*_*ijkl*_

*θ*_*ijkl*_ = *α* + *β*_*1*_ × Age_*k*_ + *β*_*2*_ × Sex_*l*_ *+ β*_*3*_ × rainfall_*ij*_ *+ β*_4_ × Tempmax_*ij*_ + u_*i*_ + s_*i*_

where E is the expected number of cases (acting as an offset to control for population size) and θ is the mean log relative risk (RR); α is the intercept, and *β*_1_*, β*_2_*, β*_3_ and *β*_4_ the coefficients for age (<5 years reference), sex (male reference), rainfall and maximum temperature, respectively; u_*i*_ is the unstructured random effect (assumed to have a mean of zero and variance σ_u_^2^) and s_*i*_ is the spatially structured random effect (assumed to have a mean of zero and variance σ_s_^2^).

Spatially structured random effect was modelled with a conditional autoregressive (CAR) prior structure. An adjacency weights matrix was used to calculate the spatial relationships between the sub-districts, a weight of “1” was assigned when two sub-districts shared a border and “0” if they did not. For the intercept, a flat prior distribution was specified, whereas a normal prior distribution was specified for the coefficients. The priors for the precision of unstructured and spatially structured random effects were specified using non-informative gamma distributions with shape and scale parameters equal to 0.01. Models were also developed without the structured and unstructured random effects to assess whether the inclusion of these components improved model fit.

An initial burn-in of 10,000 iterations was run and these iterations were discarded. Subsequent blocks of 20,000 iterations were run and examined for convergence. Convergence was assessed by visual inspection of posterior density, history plots and Gelman-Rubin statistics, and occurred at approximately 100,000 iterations for each model. Hundred thousand values from the posterior distributions of each model parameter were stored and summarised for the analysis (posterior mean and 95%CrI).

In all analyses, an α-level of 0.05 was adopted to indicate statistical significance (as indicated by 95% CrI for relative risks [RR] that excluded 1). ArcMap 10.5 software (ESRI, Redlands, CA) was used to generate maps of the posterior means of the unstructured and structured random effects and the spatiotemporal random effects obtained from the three models.

## Supplementary information

Supplemantary materials.

## Data Availability

The datasets generated during and/or analysed for this current study will be made available from the corresponding author on reasonable request.
