# Supplementary material for: A spatio-temporal analysis to identify the drivers of malaria transmission in Bhutan
Source: Sci Rep. 2020 Apr 27;10:7060. doi: 10.1038/s41598-020-63896-7 (PMC7184595; doi:10.1038/s41598-020-63896-7)
Supplement: Supplementary file 1 — Supplemantary materials. [file 41598_2020_63896_MOESM1_ESM.pdf]

A spatio-temporal analysis to identify the drivers of malaria transmission in Bhutan

Running title: Spatial and temporal analysis to identify the causes of malaria in Bhutan

Kinley Wangdi[1], Zhijing Xu[1], Apiporn T. Suwannatrai[2], Johanna Kurscheid[1], Aparna Lal[3], Rinzin Namgay[4], Kathryn Glass[3], Darren J Gray[1], Archie CA Clements[5,6]

[1]Department of Global Health, Research School of Population, Australian National University, Acton, ACT 2601, Australia

[2]Department of Parasitology, Faculty of Medicine, Khon Kaen University, Khon Kaen, Thailand

[3]National Center for Epidemiology and Population Health, Research School of Population, Australian National University, Acton, ACT 2601, Australia

[4]Vector-borne Disease Control Program, Department of Public Health, Ministry of Health, Bhutan

[5]Faculty of Health Sciences, Curtin University, Perth, WA, Australia

[6]Telethon Kids Institute, Nedlands, Australia

Corresponding author: Dr Kinley Wangdi, Department of Global Health, Research School of Population Health, Australian National University, 62 Mills Road, Acton, Canberra, ACT 2601, Australian. Email: kinley.wangdi@anu.edu.au

Supplementary Tables

Supplementary Table 1 Model comparison using Akaike's information criterion and Bayesian information criterion for *Plasmodium falciparum*

**Models**	**Observations**	**AIC**	**BIC**
**Poisson**	8,774	12,942.24	12,963.48
**ZIP**	8,774	8,627.033	8,655.35

Supplementary Table 2 Model comparison using Akaike's information criterion and Bayesian information criterion for *Plasmodium vivax*

**Models**	**Observations**	**AIC**	**BIC**
**Poisson**	8,774	13,703.93	13,725.17
**ZIP**	8,774	9,513.28	9,541.60

Supplementary Table 3 Results of Vuong test for *Plasmodium falciparum*

**Cases**	**Coefficient**	**SE**	**P value**	**95% CI**
**Rainfall lagged one month**	0.008	0.002	<0.0001	0.005, 0.011
**Maximum temperature**	0.063	0.009	<0.0001	0.045, 0.082
**Vuong test of ZIP vs standard Poisson:**			Z= 11.65	Pr > z = 0.0000

Supplementary Table 4 Results of Vuong test for *Plasmodium vivax*

**Cases**	**Coefficient**	**SE**	**P value**	**95% CI**
**Rainfall lagged one month**	0.001	0.002	<0.0001	-0.002, 0.004
**Maximum temperature**	0.085	0.008	<0.0001	0.069, 0.101
**Vuong test of ZIP vs standard Poisson:**			Z= 12.78	Pr > z = 0.0000

Supplementary Figures

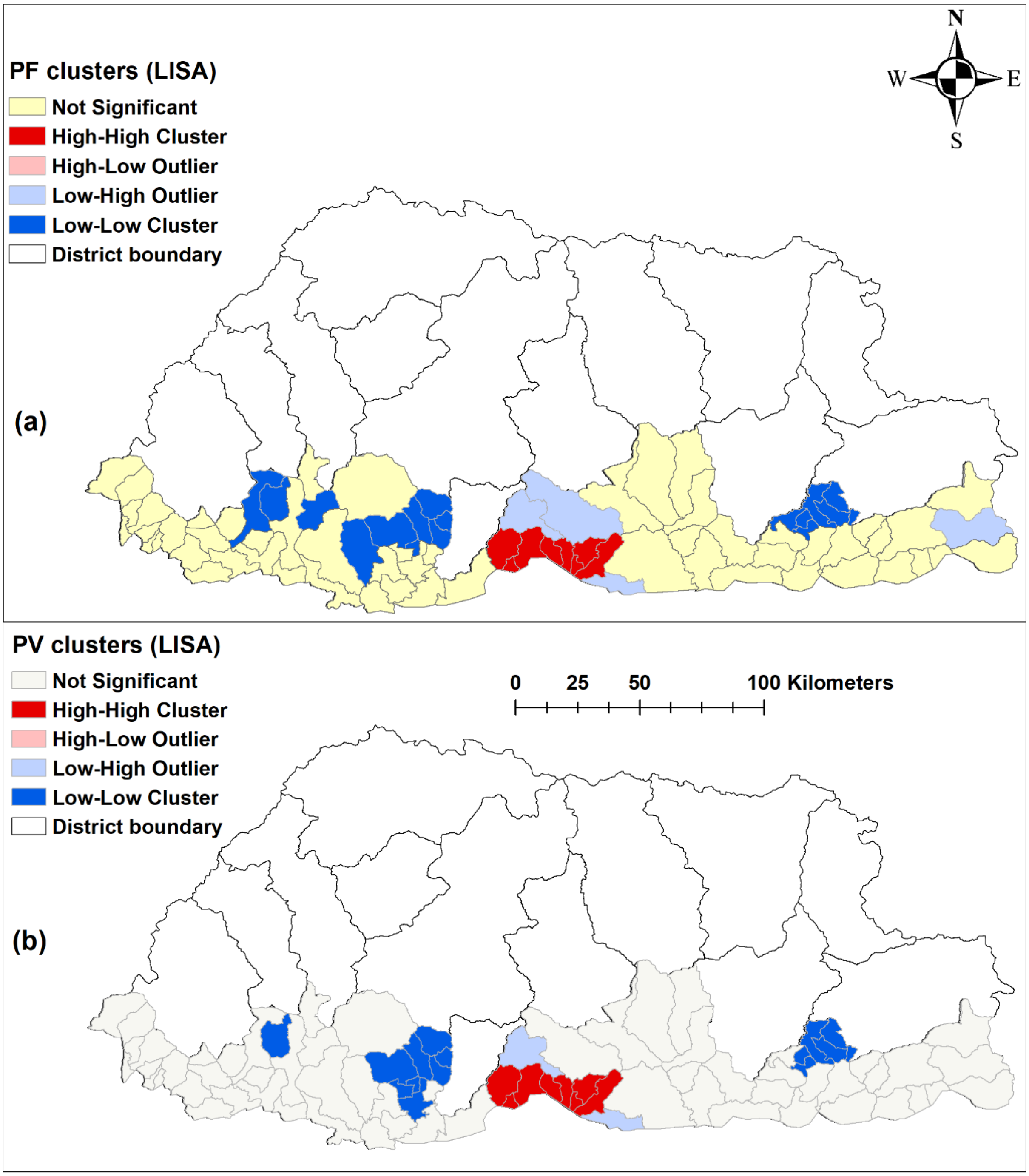

Supplementary Fig 1 a) clusters (Anselin Local Moran's I) of *Plasmodium falciparum* and b) clusters (Anselin Local Moran's I) of *Plasmodium vivax* in 82 sub-districts in seven districts of Bhutan.

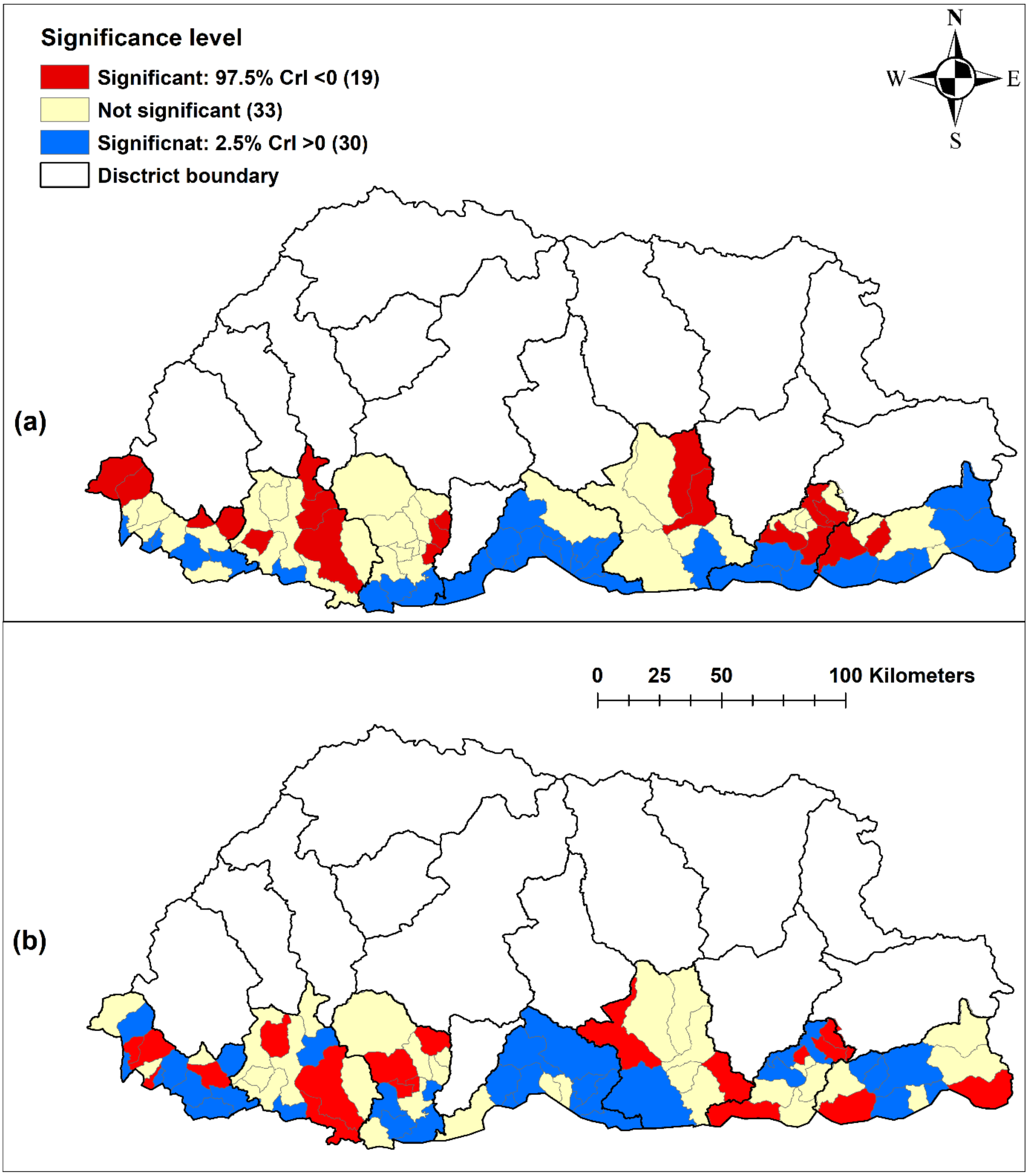

Supplementary Fig 2 Significance map of posterior mean of unstructured random effects of (a) *Plasmodium falciparum* and (b) *Plasmodium vivax* by sub-districts in Bhutan from 2006-2014.
